# Zoonotic Orthoflaviviruses Related to Birds: A Literature Review

**DOI:** 10.3390/microorganisms13071590

**Published:** 2025-07-06

**Authors:** Vladimir Savić, Ljubo Barbić, Maja Bogdanić, Ivana Rončević, Ana Klobučar, Alan Medić, Tatjana Vilibić-Čavlek

**Affiliations:** 1 Poultry Center, Croatian Veterinary Institute, 10000 Zagreb, Croatia; roncevic@veinst.hr; 2 Department of Microbiology and Infectious Diseases with Clinic, Faculty of Veterinary Medicine, University of Zagreb, 10000 Zagreb, Croatia; ljubo.barbic@vef.hr; 3 Department of Virology, Croatian Institute of Public Health, 10000 Zagreb, Croatia; maja.bogdanic@hzjz.hr (M.B.); tatjana.vilibic-cavlek@hzjz.hr (T.V.-Č.); 4 School of Medicine, University of Zagreb, 10000 Zagreb, Croatia; 5 Department of Epidemiology, Andrija Štampar Teaching Institute of Public Health, 10000 Zagreb, Croatia; ana.klobucar@stampar.hr; 6 Department of Epidemiology, Zadar County Institute of Public Health, 23000 Zadar, Croatia; alan.medic@zjz.t-com.hr

**Keywords:** orthoflavivirus, birds, zoonotic, epidemiology

## Abstract

Orthoflaviviruses (formerly flaviviruses) are known for their role in numerous diseases affecting both humans and animals. Despite the worldwide distribution of orthoflaviviruses, individual species are only found in endemic or epidemic regions. However, in recent decades, certain orthoflaviviruses have spread beyond their traditional geographic boundaries, even crossing continents. Given the long-distance movements of birds, the knowledge of zoonotic orthoflaviviruses associated with birds is essential because of their possible introduction into new regions, as was the case with West Nile virus and Usutu virus. A thorough literature review was conducted on zoonotic orthoflaviviruses related to birds, including lesser-known (re-)emerging and neglected orthoflaviviruses that are limited to specific regions and/or avian hosts but have the potential to spread to a wider geographical area and pose a higher risk of transmission to humans. Several of these viruses possess significant zoonotic potential and can cause a wide spectrum of diseases in humans, ranging from mild febrile illnesses (Zika virus) to severe neuroinvasive diseases (tick-borne encephalitis, West Nile, Japanese encephalitis virus) and hemorrhagic fevers (yellow fever, dengue virus). Geographic distribution, hosts, vectors, incidence of human infections, and impact on human and animal health of zoonotic flaviviruses related to birds are critically reviewed. The viruses have been categorized based on the role of birds as an orthoflavivirus host and the clinical presentation in human infections.

## 1. Introduction

According to the International Committee on Taxonomy of Viruses (ICTV), the genus *Flavivirus* within the family *Flaviviridae* was renamed *Orthoflavivirus* in 2023. The new name roughly translates to “true flaviviruses” or “flaviviruses sensu stricto”. Consequently, the term “orthoflavivirus” has been used for viruses of the *Orthoflavivirus* genus [1].

Viruses in the *Orthoflavivirus* genus, family *Flaviviridae* are small (50 nm) spherical enveloped viruses. Virions contain three structural proteins: capsid (C), the main envelope protein (E), and either precursor prM (immature virions), or M (mature virions). Seven nonstructural proteins (NS1, NS2A, NS2B, NS3, NS4A, NS4B and NS5) are synthesized in infected cells. The genome comprises a single long open reading frame (ORF) containing over 10,000 nucleotides that codes for all structural and nonstructural proteins. Orthoflaviviruses can infect numerous vertebrate species [2].

**Figure 1:**
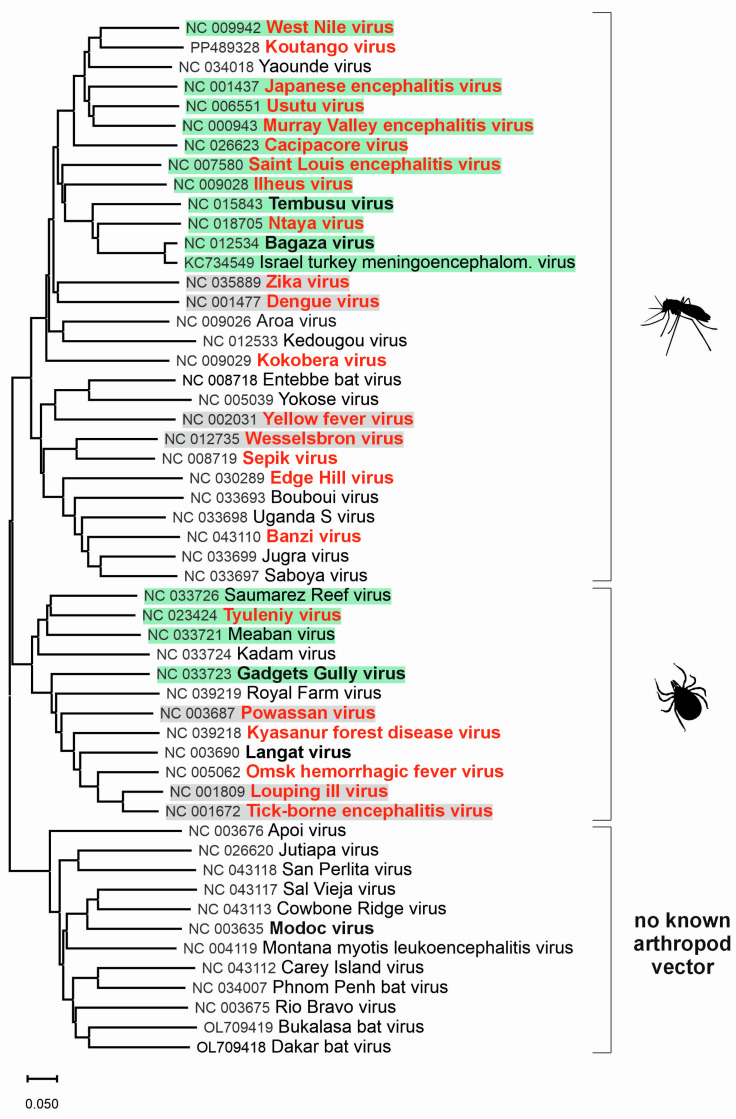
Phylogenetic tree for genus *Orthoflavivirus*. The evolutionary history was inferred from complete nucleotide sequences of *Orthoflavivirus* species according to the current taxonomy [2]. For better clarity, previous species names were used instead of binomial species names. The green background indicates viruses for which birds are the main host, and the grey background indicates other viruses related to birds. Viruses highlighted in bold red can cause clinical infections in humans, while bold black-highlighted viruses are associated with subclinical infections in humans.

While some viruses can infect and multiply in various species (mammals, birds), others infect only a limited range of vertebrate hosts such as primates [3]. Most orthoflaviviruses are arboviruses transmitted by hematophagous arthropods (vectors) to vertebrate hosts. Of the known orthoflaviviruses, almost 50% are transmitted by mosquitoes, mainly of the *Aedes* and *Culex* genera. These viruses typically infect both humans and other vertebrates, with mosquitoes acting as vectors that transmit the virus between hosts. Around 28% of orthoflaviviruses are transmitted by ticks. Tick-borne viruses circulate in a zoonotic cycle, involving mammalian hosts like rodents or deer. Humans represent incidental or “dead-end” hosts, typically becoming infected through tick bites. The remaining orthoflaviviruses are not associated with arthropod vectors. These viruses are generally found in rodents or bats and spread without any known arthropod vectors (Figure 1) [3].

More than 50% of known orthoflaviviruses have been linked to human diseases, including significant human pathogens like yellow fever virus (YFV), dengue virus (DENV), Zika virus (ZIKV), Japanese encephalitis virus (JEV), tick-borne encephalitis virus (TBEV), West Nile virus (WNV), and Usutu virus (USUV) [2]. The diseases caused by orthoflaviviruses may present with fever, arthralgia, rash, central nervous system symptoms (meningitis, encephalitis, myelitis), and hemorrhagic fever [2,4]. Many orthoflaviviruses are pathogenic for domestic or wild animals, including turkeys, pigs, horses, sheep, dogs, grouse, and muskrats, causing economically important diseases [2].

**Figure 2:**
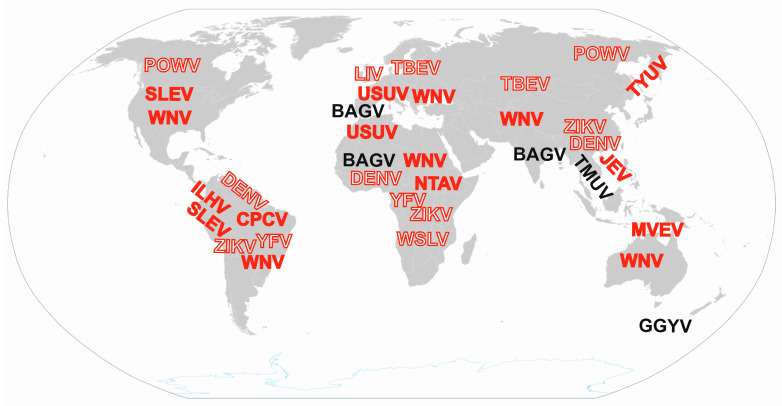
Global distribution of zoonotic orthoflaviviruses related to birds. Sporadic findings in remote locations are not indicated. Solid letters indicate viruses for which birds are the main host, while outlined letters indicate other viruses related to birds. Red highlighted viruses can cause clinical infections, while black highlighted viruses are associated with subclinical infections in humans. WNV: West Nile virus (*Orthoflavivirus nilense*), JEV: Japanese encephalitis virus (*Orthoflavivirus japonicum*), SLEV: Saint Louis encephalitis virus (*Orthoflavivirus louisense*), MVEV: Murray Valley encephalitis virus (*Orthoflavivirus murrayense*), USUV: Usutu virus (*Orthoflavivirus usutuense*), ILHV: Ilheus virus (*Orthoflavivirus ilheusense*), CPCV: Cacipacoré virus (*Orthoflavivirus cacipacoreense*), TYUV: Tyuleniy virus (*Orthoflavivirus tyuleniyense*), NTAV: Ntaya virus (*Orthoflavivirus ntayaense*), BAGV: Bagaza virus (*Orthoflavivirus bagazaense*), TMUV: Tembusu virus (*Orthoflavivirus tembusu*), GGYV: Gadgets Gully virus (*Orthoflavivirus gadgetsense*), WSLV: Wesselsbron virus (*Orthoflavivirus wesselsbronense*), POWV: Powassan virus (*Orthoflavivirus powassanense*), LIV: Louping ill virus (*Orthoflavivirus loupingi*), TBEV: Tick-borne encephalitis virus (*Orthoflavivirus encephalitidis*), DENV: Dengue virus (*Orthoflavivirus dengue*), YFV: Yellow fever virus (*Orthoflavivirus flavi*), ZIKV: Zika virus (*Orthoflavivirus zika*).

Despite the worldwide distribution of orthoflaviviruses (Figure 2), individual species are only found in endemic or epidemic regions [2]. However, in recent decades, certain orthoflaviviruses have expanded beyond their traditional geographic boundaries, even crossing continents. WNV has spread globally, resulting in human infections on every continent except Antarctica [5]. USUV, once confined to sub-Saharan Africa, is now recognized as an emerging arboviral pathogen throughout Europe [6]. The principal vertebrate hosts for WNV and USUV are birds; therefore, migratory wild birds play a crucial role in the intercontinental spread of these orthoflaviviruses [7,8]. Climate change facilitates newly introduced orthoflaviviruses to create new local spread cycles with the local vector, host, and reservoir species [9]. This calls for an awareness of other zoonotic orthoflaviviruses related to birds and their possible emergence and spread in new regions. In this article, we reviewed literature data on zoonotic orthoflaviviruses related to birds, including their geographic distribution, hosts, vectors, zoonotic potential, incidence of human infections, and impact on human and animal health. Furthermore, the orthoflaviviruses have been categorized based on the role of birds as an orthoflavivirus host and the clinical presentation in human infections.

## 2. Zoonotic Orthoflaviviruses for Which Birds Are the Main Host

### 2.1. Orthoflaviviruses Causing Clinical Infections in Humans

#### 2.1.1. West Nile Virus (*Orthoflavivirus nilense*)

WNV is a mosquito-borne orthoflavivirus of the Japanese encephalitis virus group [2], first isolated in 1937 from the blood of a febrile woman in the West Nile district of northern Uganda [10]. At least nine WNV genetic lineages have been defined, with lineages 1a and 2 responsible for the majority of human infections [11]. In nature, WNV is maintained in a mosquito-bird-mosquito cycle with mosquitoes of the *Culex* genus as the main transmitting vectors. The WNV host range is broad. It replicates in several animal species; however, only birds produce high-level viremia and are considered competent reservoirs [12,13]. Corvids (crows, blue jays, and ravens) are the most susceptible and often succumb to fatal systemic disease [13]. Infected birds frequently exhibit neurological symptoms such as impaired coordination, head tilting, tremors, weakness, signs of blindness, and lethargy, typically dying within three weeks. The Northern goshawk (*Accipiter gentilis*) is a raptor species in Europe that has been repeatedly associated with WNV infection [14]. The higher incidence in goshawks may be related to their predation of smaller birds, which can also serve as WNV reservoirs [15]. Most poultry species, such as the chicken (*Gallus gallus*), seem refractory to overt clinical disease, except for domestic goose (*Anser anser domesticus*), where WNV-associated mortality has been documented [16,17,18]. Among mammals, clinical disease is primarily exhibited in horses and humans, representing incidental or dead-end hosts [13]. WNV is the most widely distributed arbovirus worldwide, except for Antarctica [5]. In Europe, human WNV infections have increased in the past decade. In humans, an estimated 80% of infections are thought to be asymptomatic, 20% of infected individuals develop a mild febrile disease (West Nile fever), and less than 1% develop a severe form of neuroinvasive disease (WNND; meningitis, encephalitis, myelitis). Other rare presentations associated with WNV infection include retinitis [19], myocarditis [20], cerebellitis [21], polyradiculoneuritis [22], cauda equina arachnoiditis [23], and opsoclonus-myoclonus syndrome [24]. The case fatality rates in patients with WNND are up to 10% [25].

#### 2.1.2. Japanese Encephalitis Virus (*Orthoflavivirus japonicum*)

JEV is a mosquito-borne flavivirus of the Japanese encephalitis virus group [2], first isolated in 1935 from a fatal case of encephalitis in Japan [26]. According to the E gene sequence, five genotypes have been reported (GI-V) [27]. JEV is transmitted in an enzootic natural cycle between mosquitoes (mostly of the *Culex* genus) and vertebrate hosts (primarily pigs and birds) [28]. More than 90 bird species, including both domestic and wild, were found to be susceptible to JEV infection and show variable levels of viremia following natural or experimental infection [29]. Waterbirds, especially wading birds like herons and egrets, are the main source of infection [28]. Only a few bird species develop clinical signs or even die from JEV infection, while most show no clinical disease but have a high level of viremia [29]. In addition, some serosurveys have revealed that ducks and chickens are involved in the JEV epidemiological cycle, either as reservoirs or as amplifying hosts [30]. Humans represent incidental or dead-end hosts for JEV [28,31]. Endemic areas include Asia and parts of the western Pacific. However, new JEV transmission areas have emerged in several locations during the past few decades, most notably across a significantly larger area of Australia [31]. Japanese encephalitis is typically a childhood disease since most adults in endemic areas have immunity from prior JEV exposure and subclinical infections. Even though less than 1% of JEV-infected individuals develop clinical illness, the disease is often severe, with a case-fatality of up to 30% among patients with neuroinvasive infection and sequelae in 30–50% of survivors [31].

#### 2.1.3. Saint Louis Encephalitis Virus (*Orthoflavivirus louisense*)

SLEV is a mosquito-borne flavivirus of the Japanese encephalitis virus group [2], distributed throughout the Americas. The virus was first isolated in 1933 from deceased patients during the outbreak in St. Louis, Missouri, USA [32]. The birds involved in the SLEV transmission cycle include pigeons, blue jays, robins, and house sparrows found in both urban and rural environments. Mosquitoes of the genus *Culex* are vectors of SLEV. Humans and domestic animals represent dead-end hosts. Although the virus may be found in a wide geographic area ranging from Canada to Argentina, most cases occur in the eastern and central USA [33]. In the USA, from 8 to 33 human cases of SLE were reported annually to the CDC between 2014 and 2023 [34]. Most cases of SLEV infection are asymptomatic. Clinical manifestations range from non-specific febrile disease or febrile headache to aseptic meningitis and encephalitis. The case fatality rates in patients with encephalitis are 3–30%, while 5–10% experience long-term neurologic sequelae, such as gait and speech abnormalities, tremors, or behavioral problems [35]. Older age is a risk for the severe neuroinvasive forms [33].

#### 2.1.4. Murray Valley Encephalitis Virus (*Orthoflavivirus murrayense*)

MVEV is a mosquito-borne orthoflavivirus of the Japanese encephalitis virus group [2], first isolated in Murray Valley, Australia, from the brain of fatal human cases during the epidemic in 1951 [36]. MVEV is maintained in an enzootic cycle between waterbirds as natural and amplifying hosts and *Culex* mosquitoes [37]. Herons and egrets, especially the Nankeen (or rufous) night heron (*Nycticorax caledonicus*), are considered to be the main vertebrate hosts; however, members of other bird orders can also be adventitious hosts for MVEV. Despite the antibody detection in many domestic and wild animals, only rabbits and possibly western grey kangaroos develop high levels of viremia to support local transmission cycles [38]. The MVEV endemic areas are Australia and Papua New Guinea [37]. While most human infections are asymptomatic, some patients develop encephalitis. Clinical features of MVEV are similar to those of JEV and include fever, headache, confusion, and seizures (especially in children) [39]. The case fatality rate in patients with neuroinvasive disease is 15–30% [40].

#### 2.1.5. Usutu Virus (*Orthoflavivirus usutuense*)

USUV is a mosquito-borne orthoflavivirus of the Japanese encephalitis virus group [2] first isolated in 1959 from *Culex neavei* mosquitoes near the Usutu River in South Africa [41]. Eight USUV lineages have been detected so far, including three African (Africa 1–3) and five European (Europe 1–5) [42]. Although USUV is prevalent in different bird species, this orthoflavivirus is still neglected in many European countries. The USUV transmission cycle and geographic distribution overlap with that of WNV, with birds as the virus reservoirs and mosquitoes, mainly of the *Culex* genus, as vectors. Like WNV, USUV antibodies have been found in different animal species (horses, dogs, squirrels, wild boar, deer, and lizards); however, birds are considered reservoirs due to high-level viremia. Although there were no reports of bird mortality in Africa, USUV was found to be highly pathogenic to several bird species in Europe, particularly great grey owls (*Strix nebulosa*) and blackbirds (*Turdus merula*) [43]. Clinical signs of USUV infection in birds include ataxia, prostration, disorientation, and weight loss with hepatosplenomegaly as a predominant macroscopic lesion [44]. After the first human clinical cases of neuroinvasive USUV infection were reported in 2009 [45,46], the role of this virus as a zoonotic pathogen has increased in recent years. More than 100 clinical cases of human infections have been recorded in European countries, including both USUV fever and neuroinvasive diseases, often related to immunocompromised patients [47,48,49,50,51,52,53]. In addition, some atypical neurological presentations, such as idiopathic facial paralysis, were reported [54]. USUV RNA was also detected in asymptomatic blood donors [55]. Due to its neurotropism, USUV represents an increasing public health threat. Therefore, monitoring human infections and USUV circulation in migratory and resident birds and vectors is important to protect human health [56].

#### 2.1.6. Ilheus Virus (*Orthoflavivirus ilheusense*)

Ilheus virus (ILHV) is a neglected human pathogen of the Ntaya virus group [2], first isolated in 1944 from mosquitoes near Ilheus in Brazil [57,58]. The virus is endemic to Central and South America and the Caribbean [59]. ILHV is assumed to be maintained in nature by a cycle between mosquito vectors and birds. Numerous bird species belonging to several orders were found to be infected, most belonging to the Passeriformes order. However, serological surveys have also demonstrated ILHV antibodies in other vertebrates such as rodents, coatis, tortoises, water buffalo, bats, horses, sloths, monkeys, and humans. *Aedes*, *Culex*, *Coquillettidia*, *Haemagogus*, *Sabethes*, *Trichoprosopon*, *Psorophora,* and *Ochlerotatus* mosquitoes serve as vectors for ILHV, while human infection occurs via mosquito bites, especially of the genera *Psorophora* and *Ochlerotatus* [60,61]. Sporadic human infections have been reported in several countries where ILHV is endemic. Symptoms range from subclinical to severe disease; most commonly presenting with fever, headache, and myalgia, while encephalitis complicates the course of the infection in 29.4% of cases [60]. The long-term sequelae or deaths have not been described, except in a single case in 2017 with a fatal outcome in an elderly patient with encephalitis in Brazil [62]. ILHV circulation in mosquitoes, birds, humans, and other potential host species indicates its zoonotic potential comparable to that of WNV and USUV [59].

Rocio virus (ROCV) is closely related to ILHV. It was first isolated in 1975 during an outbreak of meningoencephalitis in humans in São Paulo in Brazil, causing long-term sequelae (20%) and patient deaths (10%). The epidemic lasted two years [63]. More recently, ROCV RNA was detected in two humans during dengue epidemics in Brazil (2011–2013) [64]. In addition, a serosurvey performed on Brazilian horses between 2004 and 2009 revealed that 6.1% of the collected samples tested positive for the presence of antibodies against ROCV. None of the horses examined exhibited symptoms indicative of central nervous system infections [65]. ROCV and ILHV share 73.37–74.5% amino acid identity and are considered members of the same species [66].

#### 2.1.7. Cacipacoré Virus (*Orthoflavivirus cacipacoreense*)

Cacipacoré virus (CPCV) is a mosquito-borne flavivirus of the Japanese encephalitis virus group [2] present only in Brazil. It was first isolated from a wild bird in the Amazon region in 1977 as part of research activities and was named after the Cacipacoré River in Pará State. Although CPCV was discovered nearly five decades ago, little is known about its ecology, epidemiology, genetic diversity, or clinical presentation [67,68]. According to serologic and genetic evidence of CPCV circulation, numerous mammalian hosts have been implicated in the ecology and epidemiology of CPCV; however, it is assumed that the virus is primarily transmitted between mosquitoes as vectors and birds, probably migratory species, as amplification and reservoir hosts [68,69]. *Culex* spp. is probably the main vector, but CPCV was detected in *Aedes aegypti* and *Anopheles* spp. as well [69]. The data on the bird species involved in CPCV circulation are obscure, as in most cases, the species was not determined. There is also no consensus on the bird species involved in the first CPCV detection, as it could have been the black-headed antbird (*Percnostola rufifrons*) [67,69] or black-faced antthrush (*Formicarius analis*) [68,70]. Serological surveys conducted from 1978 to 1980 have shown human exposure to CPCV [70], but the first and so far the only clinical case associated with CPCV was recorded in 2002. A 33-year-old farmer was admitted to a hospital with suspected diagnoses of both leptospirosis and yellow fever, presenting with fever, jaundice, hemorrhage, headache, myalgia, conjunctival congestion, respiratory symptoms, and renal insufficiency with a fatal outcome. CPCV virus was isolated from the patient’s serum. It is noteworthy that the post-mortem examination also serologically confirmed leptospirosis, so it is only speculative about the association of CPCV with the symptoms and the patient’s death [71,72]. Several surveillance studies conducted over the past two decades have detected horses seropositive for CPCV in peri-urban and rural areas of the Brazilian Amazon, providing strong evidence of local circulation of CPCV. Serological detection has also been observed in water buffalo and wild non-human primates in these settings [68]. The actual impact of CPCV on public health is unknown, but it has the potential to become an emerging zoonotic pathogen in South America [68].

#### 2.1.8. Tyuleniy Virus (*Orthoflavivirus tyuleniyense*)

Tyuleniy virus (TYUV) is an orthoflavivirus of the seabird tick-borne virus group formerly known as the Tyuleniy serocomplex [2]. There are only a few well-documented and/or sequenced TYUV isolates, all originating from seabird ticks (*Ixodes uriae*) found at seabird breeding sites. TYUV was initially discovered on Tyuleniy Island in the Sea of Okhotsk, Russia’s far east, in 1969 [73], followed by another isolation in Russia’s far east, the Commander Islands in the Bering Sea, in 1970 [74]. In 1986, TYUV was detected again in the Sea of Okhotsk rocky islands [75]. Studies related to TYUV isolation at the Commander Islands detected 6% TYUV seropositive residents [74]. In addition, three entomologists revealed serologically confirmed TYUV fever with lymphadenopathy, arthralgia, laryngitis, and skin petechiae during TYUV studies in Russia’s far east [76]. TYUV was also isolated in 1970 on the Pacific coast in Oregon [77] and in 1974 in Rost Island, Lofoten, Norway [78]. Several studies conducted during the 1970s have identified seropositive seabird species in Russia and North America [74,76,79,80]. Although experimental infection of a few gull species with TYUV resulted in paresis, ataxia, and death, no cases of disease caused by TYUV have been reported in free-living gulls [76]. Although TYUV does not pose a significant public health threat, it is important to monitor individuals who have been exposed to seabird ticks, especially *Ixodes uriae*, for signs of fever, as they may be at risk of TYUV infection.

#### 2.1.9. Ntaya Virus (*Orthoflavivirus ntayaense*)

Ntaya virus (NTAV) is a mosquito-borne orthoflavivirus of the Ntaya virus group [2]. The virus was first isolated in 1943 from mosquitoes collected in Uganda [81]. Mosquitoes of the *Culex* genus are considered the main vectors of NTAV [82]. Studies from Romania have shown NTAV hemagglutination-inhibiting antibodies in different migratory birds and domestic animals (cattle, sheep, goats, pigs), suggesting their potential reservoir role [83,84]. In birds, NTAV is neurotropic and results in splenomegaly, and brain, liver, heart, ovary, and lung hemorrhages [85]. Serologic evidence of NTAV in humans was confirmed in West, Central, and East African countries, especially Nigeria, Kenya, Uganda, Cameroon, Central African Republic, and Zambia [82,86]. In addition, NTAV neutralizing (NT) antibodies were detected in residents of South-East Asia (Malaya, Borneo, North Vietnam, Thailand) [87]. Human NTAV infections have been associated with fever, headache, rigors, myalgia, and weakness of the arms and legs [82,86]. Although a limited number of infections have been reported, NTAV poses a public health risk due to its potential for causing neurological manifestations in humans.

### 2.2. Orthoflviviruses Causing Subclinical Infections in Humans

#### 2.2.1. Bagaza Virus (*Orthoflavivirus bagazaense*)

Bagaza virus (BAGV) is an emerging mosquito-borne orthoflavivirus of the Ntaya virus group [2] first isolated in 1966 from *Culex* spp. mosquitoes in Bagaza district, Central African Republic [88]. It was afterwards detected in mosquitoes in several African countries [89,90,91,92,93,94], the United Arab Emirates [95], and India. Similar to USUV, BAGV has spread to Europe, where it was detected in vertebrates for the first time [96]. Following the initial detection of BAGV during a neurological disease outbreak in partridges and pheasants that resulted in mass mortality in Spain in 2010 [96], subsequent outbreaks caused by BAGV were reported in Spain, Portugal [97], and a pheasant in South Africa [98]. BAGV can be transmitted by direct contact in experimentally infected partridges [99], which raises concerns about re-emergence and efficient non-vector spread of the virus. BAGV is regarded as an emerging and re-emerging pathogen with the potential to cause infections in humans [100]. In 1996, BAGV NT antibodies were detected in serum samples of 15% of encephalitis patients in Kerala state, India [101]; however, the pathogenicity of BAGV in humans remains unknown [97].

Israel turkey meningoencephalomyelitis virus (*Orthoflavivirus israelense;* ITV) is closely related to BAGV, leading some researchers to consider them to be a single virus species [102]. It was first isolated in 1958 from turkeys in Israel [103] and later in South Africa, also from turkeys [104]. ITV causes severe neuroparalytic disease in turkeys with mortality rates of over 15–30 % [105]. There are no reports of human infection with ITV, but given its high genomic and biological similarity to BAGV, the potential for ITV to infect humans cannot be ruled out.

#### 2.2.2. Tembusu Virus (*Orthoflavivirus tembusu*)

TMUV is an emerging mosquito-borne orthoflavivirus of the Ntaya virus group [2], first isolated in 1955 from *Culex tritaeniorhynchus* mosquitoes in Malaysia [106]. The virus was mostly isolated from ducks in industrial farms. In addition to ducks, TMUV naturally infects various birds, such as geese, chickens, sparrows, and pigeons. These wild birds are likely to spread the virus to farm birds by mosquitoes (mainly of the *Culex* genus) and close contact or expectoration between animals [107]. In avian populations, TMUV causes encephalitis and neurological disorders with morbidity of up to 90%, associated with female reproductive system damage, causing a severe drop in egg production in farms [108]. The virus is distributed in China and Southeast Asia; however, since 2010 and the first major outbreak in Chinese duck farms, the risk of its spread throughout Asia and the possibility of affecting humans has increased [107]. However, only a few published studies reported the evidence of TMUV infection in the human population. A study conducted among duck farm workers (2010–2012) in China showed TMUV IgG antibodies using ELISA in 71.9% of tested individuals, while NT antibodies were detected in 34.8%. In addition, RT-PCR was positive in 47.7% of oral swabs [109]. The other study tested the NT antibody response in duck farm workers (at risk) and residents near farming areas in Central Thailand (not at risk), showing no difference in NT titers between these two groups [110]. Since 2000, new variants phylogenetically closely related to the original TMUV strain (1955), named Sitiawan virus (STWV), and Baiyangdian virus (BYDV), have been identified and reported to cause avian outbreaks [107]. The findings of STWV and BYDV have raised concerns about their possible zoonotic potential, but so far, there are no reports of human infections.

#### 2.2.3. Gadgets Gully Virus (*Orthoflavivirus gadgetsense*)

Gadgets Gully virus (GGYV) is an orthoflavivirus belonging to the mammalian tick-borne virus group [2], but it is exclusively associated with seabirds as its host. GGYV can be seen as the southern hemisphere counterpart to TYUV. The two GGYV isolates, one from 1976 and the other from 1985, were obtained from seabird ticks (*Ixodes uriae*) discovered at the penguin breeding grounds on Macquarie Island, situated between Australia and Antarctica [111,112]. In 2018, the virus RNA was once again detected in *Ixodes uriae* ticks found on a penguin at Neko Harbour, Antarctica [113]. Antibodies against GGYV have been detected in 4% of residents on Heron Island, Queensland, a seabird breeding ground. However, there have been no reported cases of illness among the seropositive residents [114]. GGYV is not considered a significant public health concern, as it has been detected primarily in uninhabited or sparsely populated areas. Its vector, which is usually located in remote seabird breeding grounds, also contributes to GGYV’s negligible impact on human populations.

## 3. Other Zoonotic Orthoflaviviruses Related to Birds

### 3.1. Orthoflaviviruses Rarely Detected in Birds

#### 3.1.1. Wesselsbron Virus (*Orthoflavivirus wesselsbronense*)

Wesselsbron virus (WSLV) is a neglected mosquito-borne orthoflavivirus of the yellow fever virus (YFV) group [2], widely distributed in Africa, including Madagascar [115]. The virus was first isolated from the blood of a febrile man and a dead lamb during an outbreak in 1955 in the Wesselsbron District, the Free State Province, South Africa [116,117]. Two genetic clades of WSLV are detected in sub-Saharan Africa: clade I strains are more common and widely distributed than clade II strains, which are limited to South Africa [118]. Although the virus was isolated in 1966 from mosquitoes in Thailand, no recent evidence suggests its presence outside Africa [119]. *Aedes* mosquitoes are the primary vectors of WSLV, with a few isolations reported from *Culex*, *Mansonia*, and *Anopheles* genera [120]. WSLV disease is an acute infection affecting sheep, goats, and cattle, leading to outbreaks of abortions and perinatal mortality in small ruminants. Wild birds are also proposed reservoir hosts [119]. The virus was discovered in 1992 in a flock of ostrich chicks after 90% died at around four months, when they are usually quite resilient. At postmortem examination, only splenomegaly was found [121]. In humans, WSLV infection usually presents as an influenza-like illness, causing a short period of fever, arthralgia, and myalgia [122]. However, occupationally exposed laboratory workers have experienced more severe neurological complications, including severe headache, memory loss, muscular spasms, abdominal discomfort, and liver tenderness [123]. Given the global distribution of the *Aedes* mosquito vectors, the possible risk of WSLV spreading outside of Africa needs more attention.

#### 3.1.2. Powassan Virus (*Orthoflavivirus powassanense*)

Powassan virus POWV is an emerging orthoflavivirus of the mammalian tick-borne virus group [2], first isolated in 1958 from a fatal pediatric encephalitis case in Ontario, Canada [124]. Two genetic lineages of POWV have been detected (I and II) in Canada, the USA, and far eastern Russia, with expansion in its geographic range [125,126]. Additionally, seroprevalence studies suggest that the virus is more widely distributed across Europe and North America than previously indicated by clinical case reports [127]. In nature, POWV is maintained between ixodid ticks and small mammals, with *Ixodes scapularis* (deer tick) being the primary vector. Large mammals and humans can be infected as incidental or dead-end hosts. POWV antibodies have been detected in many animal species [128,129]. In addition, serologic evidence of POWV in a few near passerine birds was recorded in the Hudson Valley, New York State [130] and the Trois-Rivières area, Quebec [131], utilizing neutralization and hemagglutination inhibition tests, respectively. POWV was also recovered in a young bird in the USSR (Russia) [132,133]. In humans, POWV causes a severe neuroinvasive disease with a case fatality rate between 10 and 15% and long-term neurologic sequelae in over 50% of survivors [128]. Adult patients typically present with encephalitis with headache, dysarthria, visual symptoms, paresis, ataxia, and cranial nerve dysfunction, while children often have seizures [126]. Although neuroinvasive human POWV infections remain relatively rare, the number of infections has steadily increased, highlighting the need for heightened awareness among medical professionals, especially in regions where the virus is more prevalent.

#### 3.1.3. Louping Ill Virus (*Orthoflavivirus loupingi*)

Louping ill virus (LIV) is an orthoflavivirus of the mammalian tick-borne virus group [2]. The virus was first isolated in 1929 from sheep brain in Selkirkshire, Scotland [134]. LIV is endemic in the British Isles, although cases have also been reported in Norway and Denmark. A close relative of Negishi virus, which was last isolated in Japan in the mid-20th century, LIV has also been confirmed in far eastern Russia [135]. Other closely related viruses have been isolated in Spain (Spanish sheep encephalitis virus), Turkey (Turkish sheep encephalitis virus), and Greece (Greek goat encephalitis virus), which are currently considered as distinct subtypes of LIV in the recent ICTV taxonomy [2]. *Ixodes ricinus* is the only known vector of LIV. Natural hosts of LIV include sheep, mountain hares, and red grouse. Red grouse is the only bird species known to show clinical signs of the disease, with an approximate mortality rate of up to 80%. There is a lack of evidence regarding infection in other bird species; however, natural asymptomatic LIV infection has also been confirmed in willow ptarmigan (*Lagopus lagopus lagopus*) [136]. Mountain hares also play an important role in the epidemiology of LIV. Even without detectable viremia or clinical signs, they facilitate the persistence and spread of LIV within tick populations through non-viremic transmission [137]. However, LIV primarily affects sheep, with morbidity rates between 5% and 60% in newly introduced animals in endemic areas, and high mortality in clinically affected animals. Since the first reported case of possible LIV infection in humans in 1934 [138], clinically manifested disease has occasionally been reported in humans. Most historical cases of infection have been linked to occupational exposure to infected livestock, although laboratory-acquired infections have also been reported [139]. In humans, the disease is often asymptomatic or presents as a flu-like febrile illness. In rare cases, this is followed by a second phase with neurological symptoms, which in one case was fatal [140]. Despite its zoonotic potential, public health concern is low due to the low number of clinical cases in humans and unreported human-to-human transmission.

#### 3.1.4. Tick-Borne Encephalitis Virus (*Orthoflavivirus encephalitidis*)

Tick-borne encephalitis virus (TBEV) is a zoonotic orthoflavivirus of the mammalian tick-borne virus group [2], first isolated in Russia in 1937 [141]. In addition to the classical classification of TBEV into three subtypes based on geographic distribution—the European (TBEV-Eu), Siberian (TBEV-Sib), and Far-Eastern (TBEV-FE) subtypes—recent analyses suggest the existence of up to seven TBEV subtypes [142]. TBEV is endemic in several European and Asian countries. Its geographical distribution extends from Japan to northern, central, and Eastern Europe, and cases have recently been confirmed in the United Kingdom [143]. The natural cycle of TBEV involves both tick vectors and vertebrate hosts (primarily small mammals). Transmission occurs almost exclusively via ticks of the family *Ixodidae*, which function both as biological vectors and reservoir hosts. *Ixodes ricinus* is predominant in central, northern, and eastern Europe, while *Ixodes persulcatus* is a common vector in the Baltic States, Finland, and TBEV-endemic areas of Asia [144]. In addition to vector transmission, TBEV can be transmitted to humans through the consumption of unpasteurized milk or dairy products from infected domestic ruminants [145]. TBEV infections have been serologically confirmed in various bird species, with increasing seroprevalence in recent years [146]. In a study on WNV circulation conducted in 2013 in Slovakia, TBEV RNA was detected in the brain tissue of one bird [147]. Moreover, TBEV RNA has been identified in the brain tissue, as well as in certain blood and swab samples, of experimentally infected ducks, which exhibited mild to severe acute or subacute necrotizing encephalitis [148]. However, birds are not considered reservoir hosts due to limited viral replication. Instead, their role in TBEV epidemiology is primarily the transport and dissemination of infected ticks to new regions. Recent studies indicated that birds play a significant role in the short-, medium-, and long-distance dispersal of ticks, which has important implications for human disease [149]. TBEV infection has been serologically confirmed in many mammalian species; however, clinically manifested infections have only sporadically been reported in horses and dogs [150,151]. Clinical signs in domestic ruminants are uncommon [152]; however, the virus has been detected in milk even during asymptomatic infections, raising public health concerns [153]. Humans are incidental and dead-end hosts for TBEV, with the primary route of infection being the bite from an infected tick [154]. TBEV-Eu usually causes a biphasic disease, with CNS involvement in the second phase. The course of disease caused by TBEV-Sib and TBE-FE is monophasic. The case fatality rates are 0.5–2% for TBEV-Eu and 20% for TBEV-Si. In endemic areas, vaccination is the most effective method of TBE prevention [155].

### 3.2. Orthoflaviruses Detected Serologically in Birds

#### 3.2.1. Dengue Virus (*Orthoflavivirus dengue*)

DENV is an emerging mosquito-borne orthoflavivirus of the dengue virus group [2]. The virus was first isolated in 1943 during the epidemic in Nagasaki, Japan [156]. Four DENV serotypes (DENV 1–4) circulate in tropical and subtropical areas of Africa, Asia, and Central and South America. In the endemic sylvatic cycle, DENV circulates among monkeys, while in the urban cycle, humans represent the virus reservoir, and *Aedes aegypti* and *Aedes albopictus* mosquitoes are the main vectors. In addition to monkeys which act as an effective amplifying host for enzootic DENV transmission, some studies conducted in endemic areas have detected DENV-NT antibodies in different animal species (horses, bats, buffaloes, pigs, rodents). Using ELISA, IFA, or HI test, antibodies were documented in opossums, sloths, dogs, sheep and camels. Hemagglutination-inhibiting DENV antibodies were also reported in birds, including hens, ducks, and geese [157,158,159]. However, the DENV seropositivity observed in birds should be interpreted with caution given that HI was used for the serology testing and birds are known to be reservoirs of JEV. DENV RNA was found in dogs, rodents, bats, and marsupials [159]. In humans, DENV can cause a mild febrile disease presented with rash (dengue fever), dengue hemorrhagic fever, or dengue shock syndrome which occurs in patients with preexisting dengue immunity due to previous infection with different serotypes or in children with transplacentally derived maternal antibodies.

#### 3.2.2. Yellow Fever Virus (*Orthoflavivirus flavi*)

YFV is a mosquito-borne orthoflavivirus of the yellow fever virus group [2], first isolated in 1927 in Africa, simultaneously in Ghana and Senegal [160]. In a sylvatic (jungle) cycle, the virus is maintained among monkeys by *Haemagogus* and *Aedes* mosquitoes. Semidomestic mosquitoes, which can breed both in the wild and close to human habitations, infect humans and monkeys in the intermediate cycle, which is most prevalent in Africa. Infected *Aedes aegypti* and *Aedes albopictus* transmit the YFV from human to human in an urban cycle [161,162]. The possible role of birds in the maintenance of YFV in Africa was suggested in the 1940s by the detection of YFV seropositivity in the cattle egret (*Ardea ibis*), formerly known as buff-backed heron, African barn-owl (*Tyto alba affinis*), and a Senegal kingfisher (*Halcyon senegalensis senegalensis*) [163]. However, no further studies confirmed the role of birds in YFV epidemiology. Endemic areas for YFV with intermittent epidemics include sub-Saharan Africa and tropical South America. Many people infected with YFV are asymptomatic. Fever, headache, myalgia, nausea, vomiting, and loss of appetite are typical symptoms of YF. A small percentage of patients enter a second, more toxic phase characterized by jaundice and bleeding [164]. The case fatality rate in symptomatic patients is high, varying between 10 and 86% [165].

#### 3.2.3. Zika Virus (*Orthoflavivirus zika*)

ZIKV is a mosquito-borne orthoflavivirus of the Ntaya virus group [2]. The virus was isolated in 1947 from the serum of a febrile monkey caged in the Zika Forest in Uganda. In 1948, ZIKV was isolated from *Aedes africanus* mosquitoes in the same forest [166]. Two genetic lineages of ZIKV have been identified: African and Asian. In sylvatic environments, ZIKV circulates in an enzootic cycle among non-human primates and mosquitoes (*Ae. aegypti and Ae. albopictus*). The infected mosquitoes can transmit the virus to humans, starting an epidemic cycle [167]. Only a few published articles reported the detection of ZIKV antibodies in birds. In the 1970s, ZIKV antibodies were detected in two birds in Kenya [168]. Although HI antibodies to ZIKV were detected in ducks in Indonesia, NT antibodies were not detected in ducks and other wild birds tested [169]. The geographic range of ZIKV includes tropical and subtropical areas of Africa, Asia, and the Americas. Travel-related ZIKV infections are continually reported in Europe [170]. Human ZIKV infections are, in most cases, asymptomatic. The clinical manifestations of the disease include fever, conjunctivitis, and rash. Infection in pregnant women may result in congenital ZIKV syndrome with severe birth defects, including microcephaly [171]. There are no more recent data on the possible role of birds in ZIKV transmission.

Epidemiological features representing the main vectors, principal hosts, and a short description of human and animal diseases caused by orthoflaviviruses related to birds are summarized in Table 1.

## 4. Conclusions

Twelve avian orthoflaviviruses with zoonotic potential were identified, nine of which can cause clinical symptoms in humans. WNV, JEV, SLEV, MVEV, and USUV are known to cause endemics or epidemics in humans repeatedly. On the other hand, ILHV, CPCV, TYUV, and NTAV have caused a limited number of clinical infections despite a larger number of seropositive individuals. The remaining three avian orthoflavivirus species, BAGV, TMUV, and GGYV, are known to cause only subclinical infections in humans. All avian zoonotic orthoflaviviruses, except TYUV and GGYV, exhibit a similar epidemiological pattern regarding vectors and bird hosts. This suggests that they may have the potential to emerge and spread in new regions, as was the case with WNV and USUV. Additionally, BAGV has already demonstrated the ability to spread intercontinentally from Africa to Europe and Asia. Both BAGV and TMUV are known to cause subclinical infections in humans. However, they have infected a large number of farmed birds in the Iberian Peninsula and the Far East, respectively. This increases the risk of human exposure, particularly among immunocompromised individuals who may develop symptoms, as is the case with USUV infections. The TYUV and GGYV transmitting vector is *I. uriae*, a seabird tick usually located in remote seabird breeding grounds, which makes TYUV and GGYV of negligible importance for human health.

Birds do not serve as the primary host for the other seven zoonotic orthoflaviviruses associated with avian species. WSLV, LIV, POWV, and TBEV are infrequently detected in birds. In addition to ruminants, ostriches and grouse (both non-migratory bird species) are suggested as potential hosts for WSLV and LIV, respectively. Besides serologic evidence of POWV in a few bird species, there is only one documented case of virus detection in an unspecified avian species. In contrast to WSLV, LIV, and POWV, TBEV warrants greater consideration. TBEV is associated with a higher incidence of disease in humans and highlights the role of birds in the transport and dissemination of TBEV-infected ticks to new regions. Antibodies to DENV, YFV, and ZIKV have been demonstrated in birds. However, these three viruses are adapted to humans and their transmission does not depend on other vertebrates.

## Figures and Tables

**Table 1:** Zoonotic orthoflaviviruses related to birds; their main transmitting vector, principal host, and disease that they cause in humans and animals.

Virus	Main Vector	Principal Host	Human Disease	Animal Disease
WNV	Mosquitoes *Culex* spp.	Birds	Asymptomatic infections (80%), WNV fever (20%), neuroinvasive disease (<1%; immunocompromised, elderly).	Neurological disease in certain bird species, particularly Corvids, which succumb to fatal systemic disease. Horses may develop neurological disease.
JEV	Mosquitoes *Culex* spp.	Birds, pigs	Asymptomatic infections, encephalitis (<1%, mainly in children).	Only a few bird species develop clinical signs.
SLEV	Mosquitoes *Culex* spp.	Birds	Non-specific febrile disease (febrile headache), meningitis, encephalitis (older adults).	No data reported.
MVEV	Mosquitoes *Culex* spp.	Birds, family Ardeidae	Asymptomatic infections, encephalitis, seizures (in children).	No data reported.
USUV	Mosquitoes *Culex* spp.	Birds	Asymptomatic infections, USUV fever, neuroinvasive disease (immunocompromised, elderly).	Fatal infections in blackbirds and grey owls.
ILHV	Mosquitoes multiple species	Birds, mostly Passeriformes	Sporadic infections, including fever, headache, myalgia, and encephalitis, with one fatal outcome in an elderly patient.	No data reported, multiple mammalian species were found seropositive.
CPCV	Mosquitoes mainly *Culex* spp., also *Aedes* and *Anopheles*	Migratory birds	Asymptomatic infections, single case (hemorrhage, fever, respiratory symptoms, renal insufficiency, and death).	No data reported.
TYUV	Seabird ticks *Ixodes uriae*	Seabirds	Three cases in the 1970s: TYUV fever with lymphadenopathy, arthralgia, laryngitis, and skin petechiae.	No data reported in free-living birds. Experimental infection of gulls resulted in neurological disease and death.
NTAV	Mosquitoes *Culex* spp.	Birds	Fever, headache, myalgia, weakness of arms and legs.	Splenomegaly, brain and lung hemorrhages in birds.
BAGV	Mosquitoes *Culex* spp.	Birds	Unknown or uncertain; report on 15% seropositive encephalitis patients in India.	Partridges and pheasants are highly sensitive, neurological disease with up to 30% mortality rates in partridges.
TMUV	Mosquitoes *Culex* spp.	Birds, including ducks, geese, and chickens	Unknown; reports on high seroprevalence and RT-PCR positive swabs in China.	Neurological disorders and encephalitis in birds.
GGYV	Seabird ticks *Ixodes uriae*	Penguins, possibly other seabirds	Unknown; report on 4% seropositive residents on Heron Island, Queensland.	No data reported.
WSLV	Mosquitoes *Aedes* spp.	Sheep, goats, cattle, birds	Flu-like disease, neurological symptoms in exposed laboratory workers.	Fatal infections in ostrich chicks (mortality 90%).
POWV	Tick *Ixodes scapularis*	Small mammals	Neuroinvasive disease.	No data reported.
LIV	Ticks *Ixodes ricinus*	Sheep, hares, red grouse	Asymptomatic infections, non-specific febrile disease, neuroinvasive disease (rarely).	Clinical signs only in red grouse, with mortality rates up to 80%.
TBEV	Ticks *Ixodes* spp.	Small mammals	Asymptomatic infections, neuroinvasive disease (meningitis, encephalitis).	Horses may develop neurological disease.
DENV	Mosquitoes *Aedes aegypti* *Aedes albopictus*	Monkeys (silvatic cycle); humans (urban cycle)	Asymptomatic infections, dengue fever, dengue hemorrhagic fever, dengue shock syndrome.	No data reported.
YFV	Mosquitoes *Aedes aegypti* *Aedes albopictus*	Monkeys (silvatic cycle); humans (urban cycle)	Asymptomatic infections, hemorrhagic fever.	No data reported.
ZIKV	Mosquitoes *Aedes aegypti* *Aedes albopictus*	Monkeys (silvatic cycle); humans (urban cycle)	Asymptomatic infections, febrile disease with rash, congenital infections.	No data reported.

WNV: West Nile virus (*Orthoflavivirus nilense*), JEV: Japanese encephalitis virus (*Orthoflavivirus japonicum*), SLEV: Saint Louis encephalitis virus (*Orthoflavivirus louisense*), MVEV: Murray Valley encephalitis virus (*Orthoflavivirus murrayense*), USUV: Usutu virus (*Orthoflavivirus usutuense*), ILHV: Ilheus virus (*Orthoflavivirus ilheusense*), CPCV: Cacipacoré virus (*Orthoflavivirus cacipacoreense*), TYUV: Tyuleniy virus (*Orthoflavivirus tyuleniyense*), NTAV: Ntaya virus (*Orthoflavivirus ntayaense*), BAGV: Bagaza virus (*Orthoflavivirus bagazaense*), TMUV: Tembusu virus (*Orthoflavivirus tembusu*), GGYV: Gadgets Gully virus (*Orthoflavivirus gadgetsense*), WSLV: Wesselsbron virus (*Orthoflavivirus wesselsbronense*), POWV: Powassan virus (*Orthoflavivirus powassanense*), LIV: Louping ill virus (*Orthoflavivirus loupingi*), TBEV: Tick-borne encephalitis virus (*Orthoflavivirus encephalitidis*), DENV: Dengue virus (*Orthoflavivirus dengue*), YFV: Yellow fever virus (*Orthoflavivirus flavi*), ZIKV: Zika virus (*Orthoflavivirus zika*).

## Data Availability

No new data were created or analyzed in this study. Data sharing is not applicable to this article.

## Abbreviations

The following abbreviations are used in this manuscript:

YFV	Yellow fever virus (*Orthoflavivirus flavi*)
DENV	Dengue virus (*Orthoflavivirus dengue*)
ZIKV	Zika virus (*Orthoflavivirus zika*)
TBEV	Tick-borne encephalitis virus (*Orthoflavivirus encephalitidis*)
WNV	West Nile virus (*Orthoflavivirus nilense*)
USUV	Usutu virus (*Orthoflavivirus usutuense*)
JEV	Japanese encephalitis virus (*Orthoflavivirus japonicum*)
SLEV	Saint Louis encephalitis virus (*Orthoflavivirus louisense*)
MVEV	Murray Valley encephalitis virus (*Orthoflavivirus murrayense*)
ILHV	Ilheus virus (*Orthoflavivirus ilheusense*)
CPCV	Cacipacoré virus (*Orthoflavivirus cacipacoreense*)
TYUV	Tyuleniy virus (*Orthoflavivirus tyuleniyense*)
NTAV	Ntaya virus (*Orthoflavivirus ntayaense*)
BAGV	Bagaza virus (*Orthoflavivirus bagazaense*)
ITV	Israel turkey meningoencephalomyelitis virus (*Orthoflavivirus israelense*)
TMUV	Tembusu virus (*Orthoflavivirus tembusu*)
BYDV	Baiyangdian virus
STWV	Sitiawan virus
GGYV	Gadgets Gully virus (*Orthoflavivirus gadgetsense*)
WSLV	Wesselsbron virus (*Orthoflavivirus wesselsbronense*)
POWV	Powassan virus (*Orthoflavivirus powassanense*)
LIV	Louping ill virus (*Orthoflavivirus loupingi*)
ORF	Open reading frame
CDC	Centers for Disease Control and Prevention
NT	Neutralizing
